# Supplementary material for: Ambient air pollution and urological cancer risk: A systematic review and meta-analysis of epidemiological evidence
Source: Nat Commun. 2024 Jun 15;15:5116. doi: 10.1038/s41467-024-48857-2 (PMC11180144; doi:10.1038/s41467-024-48857-2)

Ambient air pollution exposure and urological cancer risk in adults: A systematic review and meta-analysis of epidemiological evidence

Jinhui Li [1†], Zhengyi Deng [1†], Simon John Christoph Soerensen [1,2], Linda Kachuri [2,3], Andres Cardenas [2], Rebecca E. Graff [4], John T. Leppert [1,5,6], Marvin E. Langston [2*], Benjamin I. Chung [1*]

Contents

Appendix 1 Details of the search strategy.

Table S1 Newcastle-Ottawa Scale for assessing the quality of (1) cohort, (2) case-control, and (3) ecological studies in the meta-analysis.

Table S2 Meta-analyses of associations of NO_X, BC, and PM_{10} with the risk of urological cancers.

Table S3 Annual reduction in absolute number of urologic cancer cases and age standardized rate (ASR) among the top 30 countries with the most urological cancer cases.

Figure S1 Funnel plots for the meta-analysis in the pooled relationships of NO_2 (left) and $PM_{2.5}$ (right) with urological cancer types.

Figure S2 Trim-and-fill funnel plots for the meta-analysis in the pooled associations of NO_2 (left) and $PM_{2.5}$ (right) with overall urological cancer risk.

Appendix 1

Details of the search strategy

PubMed (2630#)

(“air pollution” OR “air pollutant*” OR “Air Pollution”[MeSH] OR “particular matter*” OR “particulate matter*” OR “particle*” OR “PM2.5” OR “PM10” OR “PM2.5-10” OR “Particulate Matter”[MeSH] OR “black smoke” OR “black carbon” OR “soot”[MeSH] OR “Ozone” OR “O3” OR “Ozone”[MeSH] OR “carbon monoxide” OR “CO” OR “carbon monoxide”[MeSH] OR “sulfur dioxide” OR “SO2” OR “sulfur dioxide”[MeSH] OR “nitrogen dioxide” OR “NO2” OR “nitrogen dioxide”[MeSH] OR “nitrogen oxides” OR “NOX” OR “nitrogen oxides”[MeSH])
AND
(“prostate cancer*” OR “prostate neoplasm*” OR “prostate carcinoma*” OR “prostatic cancer*” OR “prostatic neoplasm*” OR “prostatic carcinoma*” OR “prostatic neoplasms”[MeSH] OR “kidney cancer*” OR “kidney neoplasm*” OR “kidney carcinoma*” OR “renal cancer*” OR “renal neoplasm*” OR “renal carcinoma*” OR “renal cell carcinoma*” OR “renal cell cancer*” OR “renal cell neoplasm*” OR “kidney neoplasms”[MeSH] OR “bladder cancer*” OR “bladder neoplasm*” OR “bladder carcinoma*” OR “urinary bladder neoplasms”[MeSH] OR “testicular cancer*” OR “testicular neoplasm*” OR “testicular carcinoma*” OR “testicle cancer*” OR “testicle neoplasm*” OR “testicle carcinoma*” OR “testicular neoplasms”[MeSH] OR “penile cancer*” OR “penile neoplasm*” OR “penile carcinoma*” OR “penis cancer*” OR “penis neoplasm*” OR “penis carcinoma*” OR “penile neoplasms”[MeSH] OR “ureter cancer*” OR “ureter neoplasm*” OR “ureter carcinoma*” OR “ureteral cancer*” OR “ureteral neoplasm*” OR “ureteral carcinoma*” OR “ureteral neoplasms”[MeSH] OR “urethra cancer*” OR “urethra neoplasm*” OR “urethra carcinoma*” OR “urethral cancer*” OR “urethral neoplasm*” OR “urethral neoplasms”[MeSH] OR “urethral carcinoma*” OR “urinary tract cancer*” OR “urinary tract neoplasm*” OR “urinary tract carcinoma*” OR “urologic cancer*” OR “urologic neoplasm*” OR “urologic carcinoma*” OR “urological cancer*” OR “urological neoplasm*” OR “urological carcinoma*” OR “urologic neoplasms”[MeSH])
AND
(“Epidemiologic Studies"[MeSH] OR “Cohort Studies"[MeSH] OR “prospective studies”[MeSH] OR “longitudinal studies”[MeSH] OR “case-control studies”[MeSH] OR “cross-sectional studies”[MeSH] OR “Follow-Up Studies”[MeSH] OR “epidemiolog*”[tiab] OR “cohort”[tiab] OR “case-control”[tiab] OR “cross-sectional”[tiab] OR "odd ratio*" OR "odds ratio*" OR "risk ratio*" OR “relative risk*” OR “hazard ratio*” OR “rate ratio*” OR “incidence ratio*”)
NOT
(“animals”[MeSH] NOT “humans”[MeSH])

Web of Science (309#)

TOPIC=(“air pollut*” OR “particular matter*” OR “particulate matter*” OR “particle*” OR “PM2.5” OR “PM10” OR “PM2.5-10” OR “black smoke” OR “black carbon” OR “soot” OR “Ozone” OR “O3” OR “carbon monoxide” OR “sulfur dioxide” OR “SO2” OR “nitrogen dioxide” OR “NO2” OR “nitrogen oxides” OR “NOX”) AND (“prostate cancer*” OR “prostate neoplasm*” OR “prostate carcinoma*” OR “prostatic cancer*” OR “prostatic neoplasm*” OR “prostatic carcinoma*” OR “kidney cancer*” OR “kidney neoplasm*” OR

“kidney carcinoma*” OR “renal cancer*” OR “renal neoplasm*” OR “renal carcinoma*” OR “renal cell carcinoma*” OR “renal cell cancer*” OR “renal cell neoplasm*” OR “bladder cancer*” OR “bladder neoplasm*” OR “bladder carcinoma*” OR “testicular cancer*” OR “testicular neoplasm*” OR “testicular carcinoma*” OR “testicle cancer*” OR “testicle neoplasm*” OR “testicle carcinoma*” OR “penile cancer*” OR “penile neoplasm*” OR “penile carcinoma*” OR “penis cancer*” OR “penis neoplasm*” OR “penis carcinoma*” OR “ureter cancer*” OR “ureter neoplasm*” OR “ureter carcinoma*” OR “ureteral cancer*” OR “ureteral neoplasm*” OR “ureteral carcinoma*” OR “urethra cancer*” OR “urethra neoplasm*” OR “urethra carcinoma*” OR “urethral cancer*” OR “urethral neoplasm*” OR “urethral carcinoma*” OR “urinary tract cancer*” OR “urinary tract neoplasm*” OR “urinary tract carcinoma*” OR “urologic cancer*” OR “urologic neoplasm*” OR “urologic carcinoma*” OR “urological cancer*” OR “urological neoplasm*” OR “urological carcinoma*” OR “urogenital neoplasm*” OR “urogenital cancer*”) AND (“Epidemiolog*” OR “Cohort" OR “prospective” OR “longitudinal stud*” OR “case control” OR “cross sectional” OR “Follow-Up Stud*” OR "odd* ratio*" OR "risk ratio*" OR “relative risk*” OR “hazard ratio*” OR “rate ratio*” OR “incidence ratio*”) NOT (animal NOT human)

Embase (1537[#])

('air pollution' OR 'air pollutant*' OR 'air pollution'/exp OR 'air pollutant'/exp OR 'particular matter*' OR 'particulate matter*' OR 'particle*' OR 'particulate matter exposure'/exp OR 'pm2.5' OR 'pm10' OR 'pm2.5-10' OR 'particulate matter'/exp OR 'black smoke' OR 'black carbon' OR 'soot' OR 'soot'/exp OR 'ozone' OR 'o3' OR 'ozone'/exp OR 'carbon monoxide' OR ('co' AND ('oxide'/exp OR air:ti,ab,kw)) OR 'carbon monoxide'/exp OR 'sulfur dioxide' OR 'so2' OR 'sulfur dioxide'/exp OR 'nitrogen dioxide' OR 'no2' OR 'nitrogen dioxide'/exp OR 'nitrogen oxide' OR 'nox' OR 'nitrogen oxide'/exp) AND ('prostate cancer*' OR 'prostate neoplasm*' OR 'prostate carcinoma*' OR 'prostatic cancer*' OR 'prostatic neoplasm*' OR 'prostatic carcinoma*' OR 'prostatic cancer'/exp OR 'kidney cancer*' OR 'kidney neoplasm*' OR 'kidney carcinoma*' OR 'renal cancer*' OR 'renal neoplasm*' OR 'renal carcinoma*' OR 'renal cell carcinoma*' OR 'renal cell cancer*' OR 'renal cell neoplasm*' OR 'kidney cancer'/exp OR 'bladder cancer*' OR 'bladder neoplasm*' OR 'bladder carcinoma*' OR 'urinary bladder cancer'/exp OR 'urinary tract cancer'/exp OR 'testicular cancer*' OR 'testicular neoplasm*' OR 'testicular carcinoma*' OR 'testicle cancer*' OR 'testicle neoplasm*' OR 'testicle carcinoma*' OR 'testis cancer'/exp OR 'penile cancer*' OR 'penile neoplasm*' OR 'penile carcinoma*' OR 'penis cancer*' OR 'penis neoplasm*' OR 'penis carcinoma*' OR 'penis cancer'/exp OR 'ureter cancer*' OR 'ureter neoplasm*' OR 'ureter carcinoma*' OR 'ureteral cancer*' OR 'ureteral neoplasm*' OR 'ureteral carcinoma*' OR 'ureter cancer'/exp OR 'urethra cancer*' OR 'urethra neoplasm*' OR 'urethra carcinoma*' OR 'urethral cancer*' OR 'urethral neoplasm*' OR 'urethral cancer'/exp OR 'urethral carcinoma*' OR 'urinary tract cancer*' OR 'urinary tract neoplasm*' OR 'urinary tract carcinoma*' OR 'urologic cancer*' OR 'urologic neoplasm*' OR 'urologic carcinoma*' OR 'urological cancer*' OR 'urological neoplasm*' OR 'urological carcinoma*' OR 'urogenital tract tumor'/exp) AND ('epidemiology'/exp OR 'cohort analysis'/exp OR 'prospective study'/exp OR 'longitudinal study'/exp OR 'case control study'/exp OR 'cross-sectional study'/exp OR 'follow up'/exp OR 'epidemiolog*':ti,ab OR 'cohort':ti,ab OR 'case-control':ti,ab OR 'cross-sectional':ti,ab OR 'odd* ratio*' OR 'risk ratio*' OR 'relative risk*' OR 'hazard ratio*' OR 'rate ratio*' OR 'incidence ratio*') NOT ('animal'/exp NOT 'human'/exp) NOT ([conference abstract]/lim OR [conference paper]/lim OR [conference review]/lim OR [preprint]/lim)

Cumulative Index to Nursing and Applied Health Literature (264#)

(“air pollution” OR “air pollutant*” OR MH “Air Pollution” OR MH “air pollutants” OR “particular matter*” OR “particulate matter*” OR “particle*” OR “PM2.5” OR “PM10” OR “PM2.5-10” OR MH“Particulate Matter” OR “black smoke” OR “black carbon” OR “soot” OR “Ozone” OR “O3” OR MH “Ozone” OR “carbon monoxide” OR “CO” OR MH“carbon monoxide” OR “sulfur dioxide” OR “SO2” OR MH “sulfur dioxide” OR “nitrogen dioxide” OR “NO2” OR MH “nitrogen dioxide” OR “nitrogen oxides” OR “NOX” OR MH “nitrogen oxides”)
AND
(“prostate cancer*” OR “prostate neoplasm*” OR “prostate carcinoma*” OR “prostatic cancer*” OR “prostatic neoplasm*” OR “prostatic carcinoma*” OR MH “prostatic neoplasms” OR “kidney cancer*” OR “kidney neoplasm*” OR “kidney carcinoma*” OR “renal cancer*” OR “renal
neoplasm*” OR “renal carcinoma*” OR “renal cell carcinoma*” OR “renal cell cancer*” OR “renal cell neoplasm*” OR MH “kidney neoplasms” OR “bladder cancer*” OR “bladder neoplasm*” OR “bladder carcinoma*” OR MH “bladder neoplasms” OR “testicular cancer*” OR “testicular neoplasm*” OR “testicular carcinoma*” OR “testicle cancer*” OR “testicle neoplasm*” OR “testicle carcinoma*” OR MH “testicular neoplasms” OR “penile cancer*” OR “penile neoplasm*” OR “penile carcinoma*” OR “penis cancer*” OR “penis neoplasm*” OR “penis carcinoma*” OR MH “penile neoplasms” OR “ureter cancer*” OR “ureter
neoplasm*” OR “ureter carcinoma*” OR “ureteral cancer*” OR “ureteral neoplasm*” OR “ureteral carcinoma*” OR MH “ureteral neoplasms” OR “urethra cancer*” OR “urethra neoplasm*” OR “urethra carcinoma*” OR “urethral cancer*” OR “urethral neoplasm*” OR MH “urethral neoplasms” OR “urethral carcinoma*” OR “urinary tract cancer*” OR “urinary tract neoplasm*” OR “urinary tract carcinoma*” OR “urologic cancer*” OR “urologic neoplasm*” OR “urologic carcinoma*” OR “urological cancer*” OR “urological neoplasm*” OR “urological carcinoma*” OR MH “urologic neoplasms” OR MH “urogenital neoplasms”)
AND
(MH “Epidemiological Research” OR “Cohort Stud*" OR MH “prospective studies” OR “longitudinal stud*” OR MH “case control studies” OR MH “cross sectional studies” OR “Follow-Up Stud*” OR TI (“epidemiolog*” OR “cohort” OR “case-control” OR “cross-sectional”) OR AB (“epidemiolog*” OR “cohort” OR “case-control” OR “cross-sectional”) OR "odd* ratio*" OR "risk ratio*" OR “relative risk*” OR “hazard ratio*” OR “rate ratio*” OR “incidence ratio*”)
NOT (MH “animals” NOT MH “humans”)

Scopus (599#)

TITLE-ABS-KEY (“air pollut*” OR “particular matter*” OR “particulate matter*” OR “particle*” OR “PM2.5” OR “PM10” OR “PM2.5-10” OR “black smoke” OR “black carbon” OR “soot” OR “Ozone” OR “O3” OR “carbon monoxide” OR “sulfur dioxide” OR “SO2” OR “nitrogen dioxide” OR “NO2” OR “nitrogen oxides” OR “NOX”)
AND TITLE-ABS-KEY (“prostate cancer*” OR “prostate neoplasm*” OR “prostate carcinoma*” OR “prostatic cancer*” OR “prostatic neoplasm*” OR “prostatic carcinoma*” OR “kidney cancer*” OR “kidney neoplasm*” OR “kidney carcinoma*” OR “renal

cancer*” OR “renal neoplasm*” OR “renal carcinoma*” OR “renal cell carcinoma*” OR “renal cell cancer*” OR “renal cell neoplasm*” OR “bladder cancer*” OR “bladder neoplasm*” OR “bladder carcinoma*” OR “testicular cancer*” OR “testicular neoplasm*” OR “testicular carcinoma*” OR “testicle cancer*” OR “testicle neoplasm*” OR “testicle carcinoma*” OR “penile cancer*” OR “penile neoplasm*” OR “penile carcinoma*” OR “penis cancer*” OR “penis neoplasm*” OR “penis carcinoma*” OR “ureter cancer*” OR “ureter neoplasm*” OR “ureter carcinoma*” OR “ureteral cancer*” OR “ureteral neoplasm*” OR “ureteral carcinoma*” OR “urethra cancer*” OR “urethra neoplasm*” OR “urethra carcinoma*” OR “urethral cancer*” OR “urethral neoplasm*” OR “urethral carcinoma*” OR “urinary tract cancer*” OR “urinary tract neoplasm*” OR “urinary tract carcinoma*” OR “urologic cancer*” OR “urologic neoplasm*” OR “urologic carcinoma*” OR “urological cancer*” OR “urological neoplasm*” OR “urological carcinoma*” OR “urogenital neoplasm*” OR “urogenital cancer*”)
AND
TITLE-ABS-KEY (“Epidemiolog*” OR “Cohort" OR “prospective” OR “longitudinal stud*” OR “case control” OR “cross sectional” OR “Follow-Up Stud*” OR "odd* ratio*" OR "risk ratio*" OR “relative risk*” OR “hazard ratio*” OR “rate ratio*” OR “incidence ratio*”)
AND NOT TITLE-ABS-KEY (animal NOT human)

Cochrane Library (1#)

(“air pollut*” OR “particular matter*” OR “particulate matter*” OR “particle*” OR “black smoke” OR “black carbon” OR “soot” OR “Ozone” OR “carbon monoxide” OR “sulfur dioxide” OR “nitrogen dioxide” OR “nitrogen oxides”) AND (“prostate cancer*” OR “prostate neoplasm*” OR “prostate carcinoma*” OR “prostatic cancer*” OR “prostatic neoplasm*” OR “prostatic carcinoma*” OR “kidney cancer*” OR “kidney neoplasm*” OR “kidney carcinoma*” OR “renal cancer*” OR “renal neoplasm*” OR “renal carcinoma*” OR “renal cell carcinoma*” OR “renal cell cancer*” OR “renal cell neoplasm*” OR “bladder cancer*” OR “bladder neoplasm*” OR “bladder carcinoma*” OR “testicular cancer*” OR “testicular neoplasm*” OR “testicular carcinoma*” OR “testicle cancer*” OR “testicle neoplasm*” OR “testicle carcinoma*” OR “penile cancer*” OR “penile neoplasm*” OR “penile carcinoma*” OR “penis cancer*” OR “penis neoplasm*” OR “penis carcinoma*” OR “ureter cancer*” OR “ureter neoplasm*” OR “ureter carcinoma*” OR “ureteral cancer*” OR “ureteral neoplasm*” OR “ureteral carcinoma*” OR “urethra cancer*” OR “urethra neoplasm*” OR “urethra carcinoma*” OR “urethral cancer*” OR “urethral neoplasm*” OR “urethral carcinoma*” OR “urinary tract cancer*” OR “urinary tract neoplasm*” OR “urinary tract carcinoma*” OR “urologic cancer*” OR “urologic neoplasm*” OR “urologic carcinoma*” OR “urological cancer*” OR “urological neoplasm*” OR “urological carcinoma*” OR “urogenital neoplasm*” OR “urogenital cancer*”)

China National Knowledge Infrastructure (CNKI) (41#)
(TKA='空气污染' OR TKA='大气污染' OR TKA='颗粒' OR TKA='二氧化硫' or TKA='二氧化氮' or TKA='一氧化碳' or TKA='臭氧' or TKA='黑炭') and (TKA='前列腺癌' or TKA='前列腺肿瘤' or TKA='膀胱癌' or TKA='膀胱肿瘤' or TKA='肾癌' or TKA='肾肿瘤' or TKA='肾细胞癌' or TKA='睾丸癌' or TKA='睾丸肿瘤' or TKA='尿道癌' or TKA='尿道肿瘤' or TKA='输尿管癌' or TKA='输尿管肿瘤' or TKA='泌尿系统癌症' or TKA='泌尿系统肿瘤') and (TKA='流行病' or TKA='队列研究' or TKA='病例对照研究' or TKA='横断面研究')

Wanfang Data (41#)

全部:(“空气污染” OR “大气污染” OR “PM” OR “颗粒” OR “二氧化硫” OR “二氧化氮” OR “一氧化碳” OR “臭氧” OR “黑炭”) AND 全部:(“前列腺癌” OR “前列腺肿瘤” OR “膀胱癌” OR “膀胱肿瘤” OR “肾癌” OR “肾肿瘤” OR “肾细胞癌” OR “睾丸癌” OR “睾丸肿瘤” OR “尿道癌” OR “尿道肿瘤” OR “输尿管癌” OR “输尿管肿瘤” OR “泌尿系统癌症” OR “泌尿系统肿瘤”) AND 全部:(“流行病” OR “队列研究” OR “病例对照研究” OR “横断面研究”)

Notes: # number of the search results for each electronic database

Table S1. Newcastle-Ottawa Scale for assessing the quality of (1) cohort, (2) case-control, and (3) ecological studies in the meta-analysis.

**Table S1.1**									
	**Selection**				**Comparability**	**Outcome**			
Cohort studies (Year) [citation]	Representativeness of the exposed cohort[^]	Selection of the non-exposed cohort	Ascertainment of exposure [*]	Demonstration that outcome of interest was not present at start of study [#]	Comparability of cohorts on the basis of the design or analysis [&]	Assessment of outcome	Was follow-up long enough for outcomes to occur [$]	Adequacy of follow up of cohorts	Quality score (0-9)
**For systematic review and meta-analysis**									
Raaschou-Nielsen (2011) [38]	*	*	*	*	**	*	*	*	9
Raaschou-Nielsen (2017) [39]	*	*	*	*	**	*	*	*	9
Turner (2017) [42]	*	*	*		**	*	*		8
Cohen (2018) [33]		*	*	*	**	*	*	*	8
Datzmann (2018) [35]	*	*	*	*		*	*		6
Pedersen (2018) [37]	*	*	*	*	**	*	*	*	9
Gandini (2018) [36]	*	*	*	*	**	*	*	*	9
Coleman (2020) [34]	*	*	*		**	*	*	*	8
Chen (2022) [32]	*	*	*	*	**	*	*	*	9
Huang (2022) [31]	*	*	*	*	**	*	*	*	9
Shin (2022) [30]	*	*	*		**	*	*	*	8
Hvidtfeldt (2022) [45]	*	*	*	*	**	*	*	*	9
**For systematic review only**									
Ancona (2015) [@ 48]		*	*			*	*	*	5
Wong (2016) [49]	*	*	*		**	*	*	*	8
Cohen (2017) [52]		*	*	*	**	*	*	*	8
Park (2023) [61]	*	*		*	**	*	*		7
Lim (2023) [62]		*	*	*	**	*	*	*	8
Wei (2023) [60]	*	*	*	*	**	*	*	*	9

**Table S1.2**									
	**Selection**				**Comparability**	**Exposure**			
Case-control studies [citation]	Case definition adequate	Representativeness of the cases	Selection of controls	Definition of controls	Comparability of cohorts on the basis of the design or analysis [&]	Assessment of exposure[*]	Same method of ascertainment for cases and controls	Non-response rate [¶]	Quality score (0-9)
**For systematic review and meta-analysis**									

Shekarrizfard (2018) [40]	*	*	*	*	**	*	*	*	9
Turner (2019) [43]	*			*	**	*	*	*	8
Taj (2022) [41]	*	*	*	*		*	*	*	7
Youogo (2022) [44]	*	*	*	*	**	*	*		8
Felici (2024) [47]	*	*	*	*		*	*	*	7
**For systematic review only**									
Liu (2009) [54]	*	*	*	*			*		5
Parent (2013) [58]	*	*	*	*	**	*	*	*	9
Shekarrizfard (2015) [56]	*	*	*	*	**	*	*	*	9
Weichenthal (2017) [57]	*	*	*	*	**	*	*		8
Dummer (2023) [63]	*	*	*			*	*		5

**Table S1.3**						
	**Selection**			**Comparability**	**Outcome**	
Ecological Studies [citation]	Representativeness of the study population^	Ascertainment of exposure*	Exposure measured before outcome (lagged exposure)	Comparability of cohorts on the basis of the design or analysis&	Assessment of outcome	Quality score (0-6)
**For systematic review and meta-analysis**						
Coleman (2020) (2) [46]	*	*	*	**	*	6
Yu (2022) [28]	*	*	*		*	4
Yu (2022) (2) [27]	*	*	*		*	4
Fan (2023) [29]	*	*	*		*	4
**For systematic review only**						
Yanaji (2012) [59]	*		*		*	3
Al-Ahmadi (2013) [50]	*	*			*	3
Yeh (2017) [51]	*	*		**	*	5
Collarile (2017) [@ 53]		*			*	2
Wang (2019) [55]	*	*			*	3

Notes:

^ 1 star if the exposure level in the study population can represent environmental air pollution in the source population.

*1 star if the study used methods beyond air monitors to measure the air pollution concentration.

#1 star if the study removed participants who were diagnosed with cancer before enrolment.

&1 star if the study accounted for age, sex, and smoking history in statistical analysis, and 2 stars if the study accounted for additional variables.

$1 star if the study followed participants >5 years.

¶1 star if the response rate between cases and controls differed by >5%

@The environmental air pollutants included the source of nearby incinerators or coal-fired and oil-thermal power plants.

Table S2. Meta-analyses of associations of NO_X, BC, PM_{10} and O_3 with the risk of urological cancers.

Pollutant	UCa Type (number of associations)	Summary RR	95%CI	I^2 (%)
NOx	Overall (10)	1.01	0.99,1.03	36.21
	BCa (4)	1.02	1.00.1.05	13.38
	KCa (3)	1.01	0.99,1.03	0.02
	PCa (3)	0.99	0.98,1.01	0.08
BC	Overall (10)	1.00	0.92,1.09	0.00
	BCa (5)	1.00	0.84,1.19	0.00
	KCa (4)	1.11	0.78,1.57	12.49
PM_{10}	Overall (9)	1.13	0.88,1.44	15.86
	BCa (3)	0.89	0.45,1.73	78.99
	KCa (3)	1.04	0.84,1.29	0.00
	PCa (3)	1.14	1.02,1.28	34.41
O_3*	Overall (6)	1.00	0.94,1.07	0.02

Notes:
* Only the overall RR was calculated as the number of associations for each type of UCa is less than 2.
Abbreviations: BCa, bladder cancer; BC, black carbon; CI, confidence interval; KCa, kidney cancer; NOx, nitrogen oxides; PCa, prostate cancer; RR, relative risk; PM_{10}, particular matter with a diameter of 10 micrometres or less; UCa, urological cancer.

Table S3. Annual reduction in absolute number of urologic cancer cases and age standardized rate (ASR) among the top 30 countries with the highest $PM_{2.5}$ exposure.

Country	$PM_{2.5}$ (μg/m^3)	Prostate and testicular cancer (males)			Kidney and bladder cancer (males and females)			All urological cancer	
		Number of cases	ASR (per 100000)	ASR reduction	Number of cases	ASR (per 100000)	ASR reduction	Number of cases	Number reduction
Egypt	63.16	5039	24.1	12.1	9602	42.6	21.5	14641	7376
Nigeria	55.64	16173	59.3	27.0	1873	6.8	3.1	18046	8230
India	50.17	38786	10.1	4.2	25795	6.5	2.7	64581	27025
China	38.15	119286	17.8	5.8	113353	17.0	5.6	232639	75952
Iran	31.62	9438	37.0	10.0	5246	20.5	5.5	14684	3973
Thailand	24.64	8847	25.2	5.2	5000	14.2	2.9	13847	2847
South Korea	24.04	14193	47.4	9.5	8070	28.4	3.6	22263	3159
Turkey	23.25	20861	75.9	14.6	13664	49.9	9.6	34525	6629
South Africa	19.75	13602	116.3	18.2	2266	18.7	2.9	15868	2487
Indonesia	19.34	14765	20.7	3.2	8089	10.5	1.6	22854	3484
Poland	18.83	19307	88.8	13.1	12235	56.7	8.3	31542	4642
Mexico	17.83	29766	77.6	10.6	6159	16.2	2.2	35925	4910
Czechia	14.34	9587	151.1	15.0	4337	66.0	6.5	13924	1380
Italy	14.22	41564	112.5	11.0	30134	66.7	6.5	71698	7009
Colombia	14.04	15711	90.2	8.6	2779	16.1	1.5	18490	1771
Ukraine	13.51	11959	59.2	5.3	7994	41.5	3.7	19953	1794
Romania	13.30	8451	75.4	6.6	5861	55.9	4.9	14312	1253
Argentina	12.04	13624	83.4	6.1	6297	38.5	2.8	19921	1462
Brazil	10.94	100173	133.9	8.2	18324	24.0	1.5	118497	7213
Japan	10.84	108371	92.1	5.5	46131	42.4	2.5	154502	9227
Netherlands	10.74	15365	138.4	8.1	7647	64.2	3.8	23012	1348

Germany	10.73	72293	125.3	7.3	36424	59.6	3.5	108717	6355
France	10.46	68657	178.6	9.9	22940	53.6	3.0	91597	5069
United Kingdom	9.52	59189	140.8	6.3	17496	39.8	1.8	76685	3407
Spain	9.34	35635	125.0	5.3	20828	67.5	2.9	56463	2390
Australia	8.93	17845	131.3	4.9	5708	39.5	1.5	23553	884
Russian Federation	8.88	48974	78.0	2.9	27511	45.0	1.7	76485	2825
United States of America	7.18	218211	128.3	2.1	105257	57.2	1.0	323468	5408
Canada	6.39	31104	143.3	1.0	12002	50.6	0.4	43106	310
Sweden	5.96	11304	178.5	0.3	3177	45.7	0.1	14481	28

Notes:
Abbreviations: $PM_{2.5}$, fine inhalable particles, with diameters that are generally 2.5 micrometers and smaller; ASR, age standardized rate.

Figure S1. Funnel plots for the meta-analysis in the pooled relationships of NO_2 (left) and $PM_{2.5}$ (right) with urological cancer types. For NO_2 n=28 association estimates; For $PM_{2.5}$ n=41 association estimates.

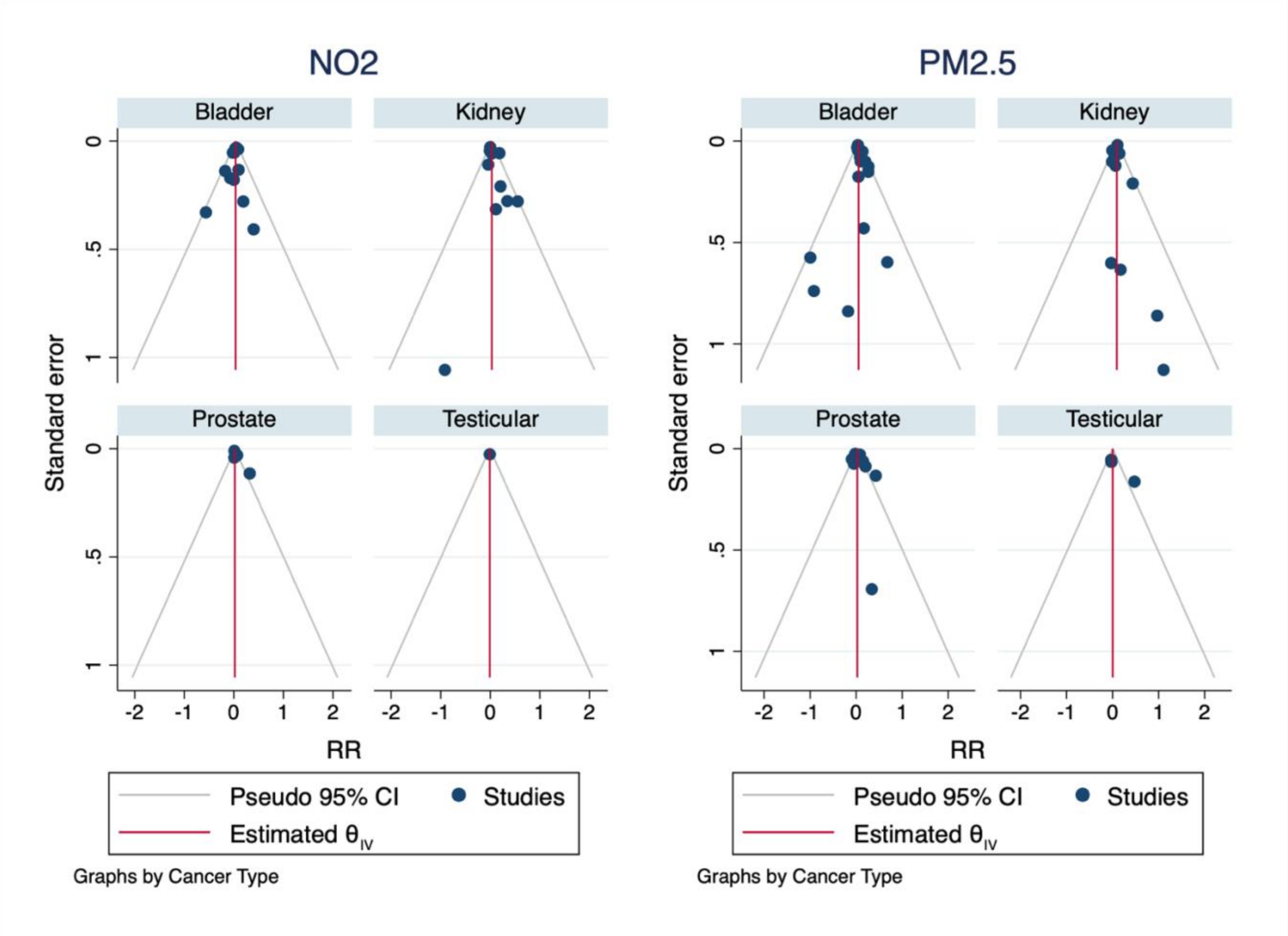

Figure S2. Trim-and-fill funnel plots for the meta-analysis in the pooled associations of NO_2 (left) and $PM_{2.5}$ (right) with overall urological cancer risk. For NO_2 n=30 association estimates; For $PM_{2.5}$ n=46 association estimates.

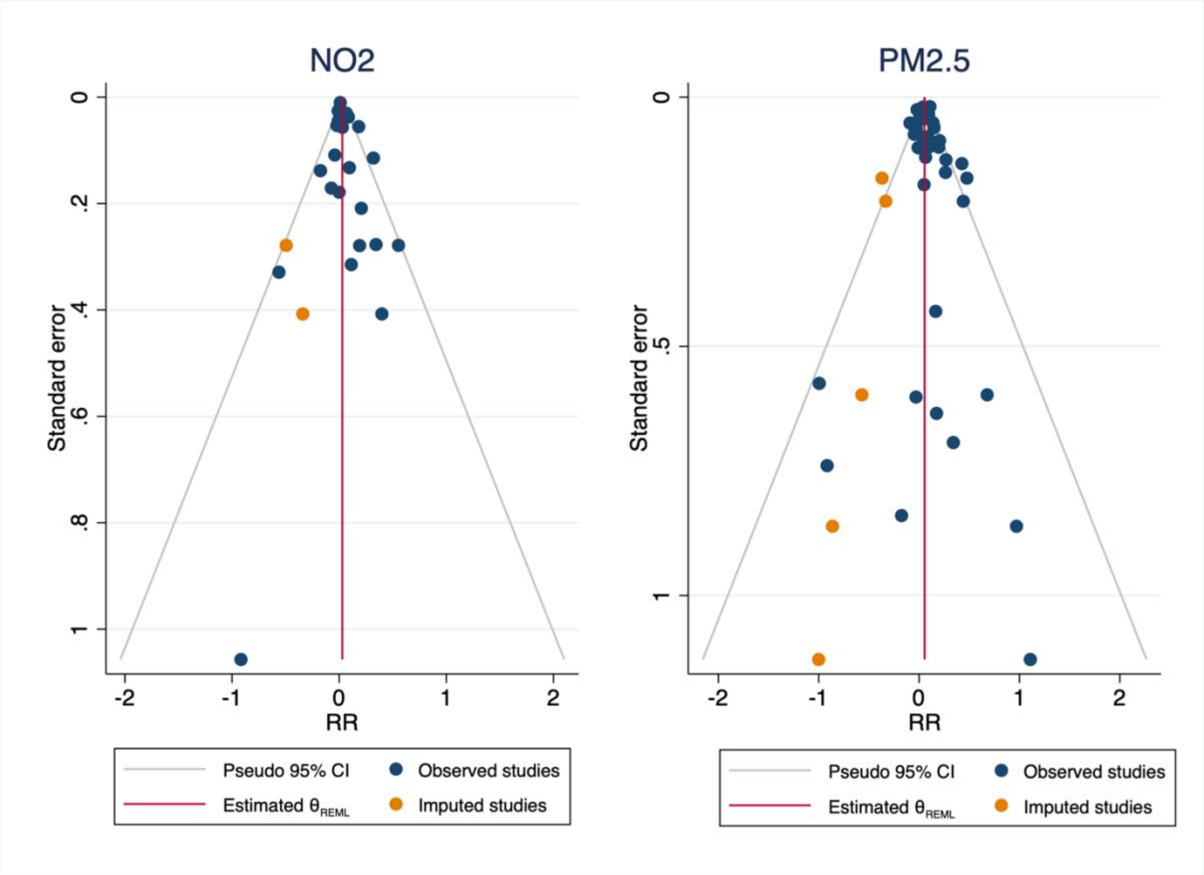

Supplement: Supplementary file 1 — Supplementary Information [file 41467_2024_48857_MOESM1_ESM.pdf]
